# Supplementary material for: Bioconjugation strategy for cell surface labelling with gold nanostructures designed for highly localized pH measurement
Source: Nat Commun. 2018 Dec 11;9:5278. doi: 10.1038/s41467-018-07726-5 (PMC6290020; doi:10.1038/s41467-018-07726-5)
Supplement: Supplementary file 2 — Supplementary Information [file 41467_2018_7726_MOESM2_ESM.pdf]

Supplementary information

Bioconjugation strategy for cell surface labelling with gold nanostructures designed for highly localized pH measurement

Leonardo Puppulin[1], Shigekuni Hosogi[1,2], Hongxin Sun[3], Kazuhiko Matsuo[4], Toshio Inui[5,6], Yasuaki Kumamoto[7], Toshinobu Suzaki[8], Hideo Tanaka[7] and Yoshinori Marunaka[1, 5,9]

[1]*Department of Molecular Cell Physiology, Graduate School of Medical Science, Kyoto Prefectural University of Medicine, Kajii-cho, Kawaramachi-Hirokoji, Kyoto 602-8566, Japan*

[2]*Department of Clinical and Translational Physiology, Kyoto Pharmaceutical University, 5 Nakauchi-cho, Misasagi, Yamashina-ku, Kyoto 607-8414, Japan*

[3]*College of Pharmaceutical Sciences, Ritsumeikan University, 1-1-1 Nojihigashi, Kusatsu, Shiga, 525-8577, Japan*

[4]*Department of Anatomy and Developmental Biology, Graduate School of Medical Science, Kyoto Prefectural University of Medicine, Kajii-cho, Kawaramachi-Hirokoji, Kyoto 602-8566, Japan*

[5]*Research Center for Drug Discovery and Pharmaceutical Development Science, Research Organization of Science and Technology, Ritsumeikan University, Kusatsu 525-8577, Japan*

[6]*Saisei Mirai Clinics, Moriguchi, 3-34-8 Okubocho, Moriguchi-shi, Osaka 570-0012, Japan*

[7]*Department of Pathology and Cell Regulation, Graduate School of Medical Science, Kyoto Prefectural University of Medicine, Kajii-cho, Kawaramachi-Hirokoji, Kyoto 602-8566, Japan*

[8]*Department of Biology, Graduate School of Science, Kobe University, 1-1 Rokkodai-cho, Nada-ku, Kobe 657-8501, Japan*

[9]*Research Institute for Clinical Physiology, Kyoto Industrial Health Association, 67 Kitatsuboi-cho, Nishino-kyo, Nakagyo-ku, Kyoto 604-8472, Japan*

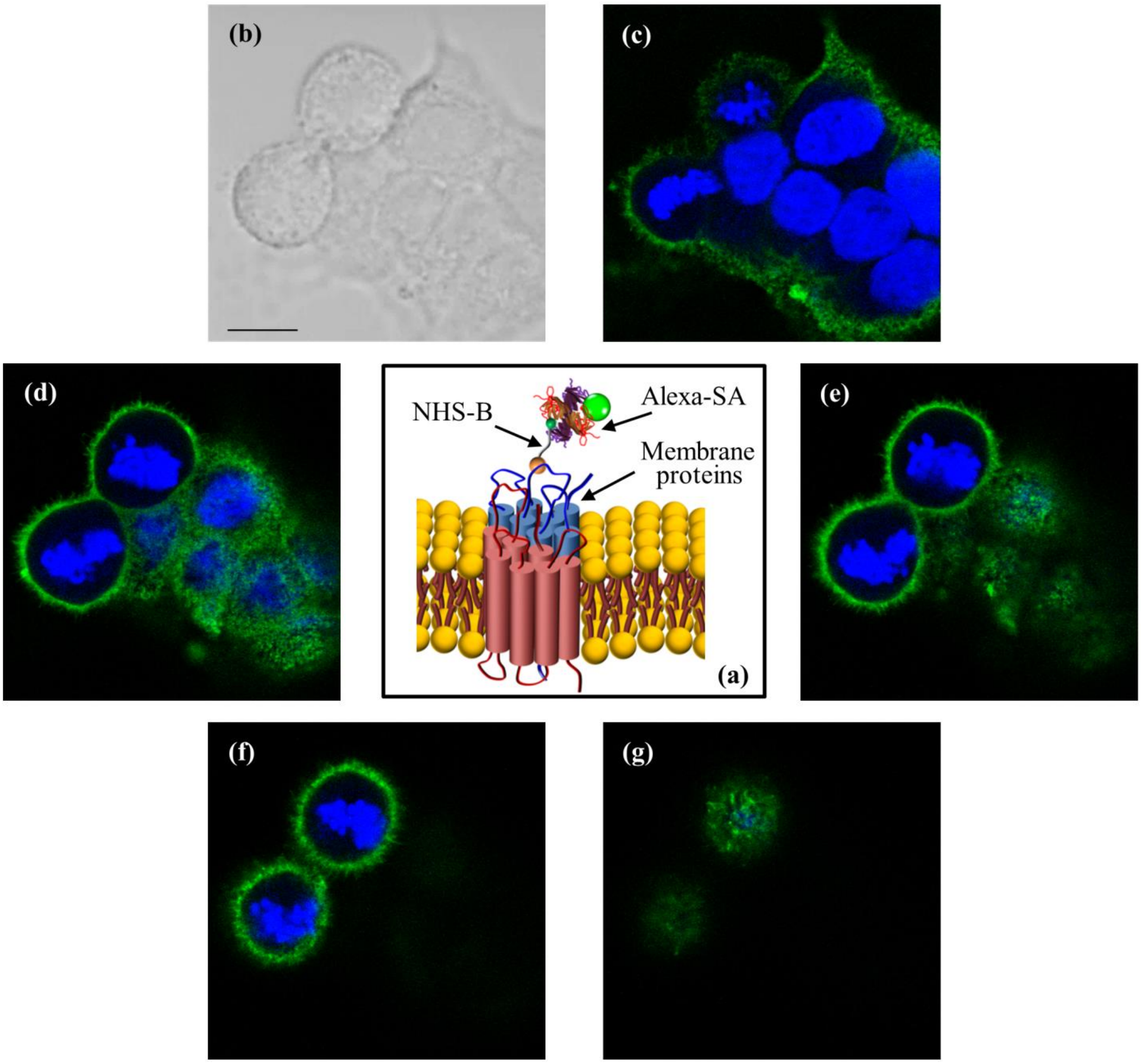

Supplementary Figure 1: Confirmation of cell surface labelling by NHS-B.

CFM analysis was performed on MKN28 cells after treatment in isotonic buffer solution at pH 8 with NHS-B functionalized with Alexa-SA, as shown in the sketch of (a). The confocal fluorescence images collected from the cell cluster in (b) are shown at different positions of the focal plane: z= 3 µm (c), 6 µm (d), 10 µm (e), 13 µm (f) and 18 (g) µm (z=0 is the position of the glass bottom dish). In Supplementary Movie 2, we combined the collected images to create a movie of the z-stack sections through the cells. Scale bar: 10 µm.

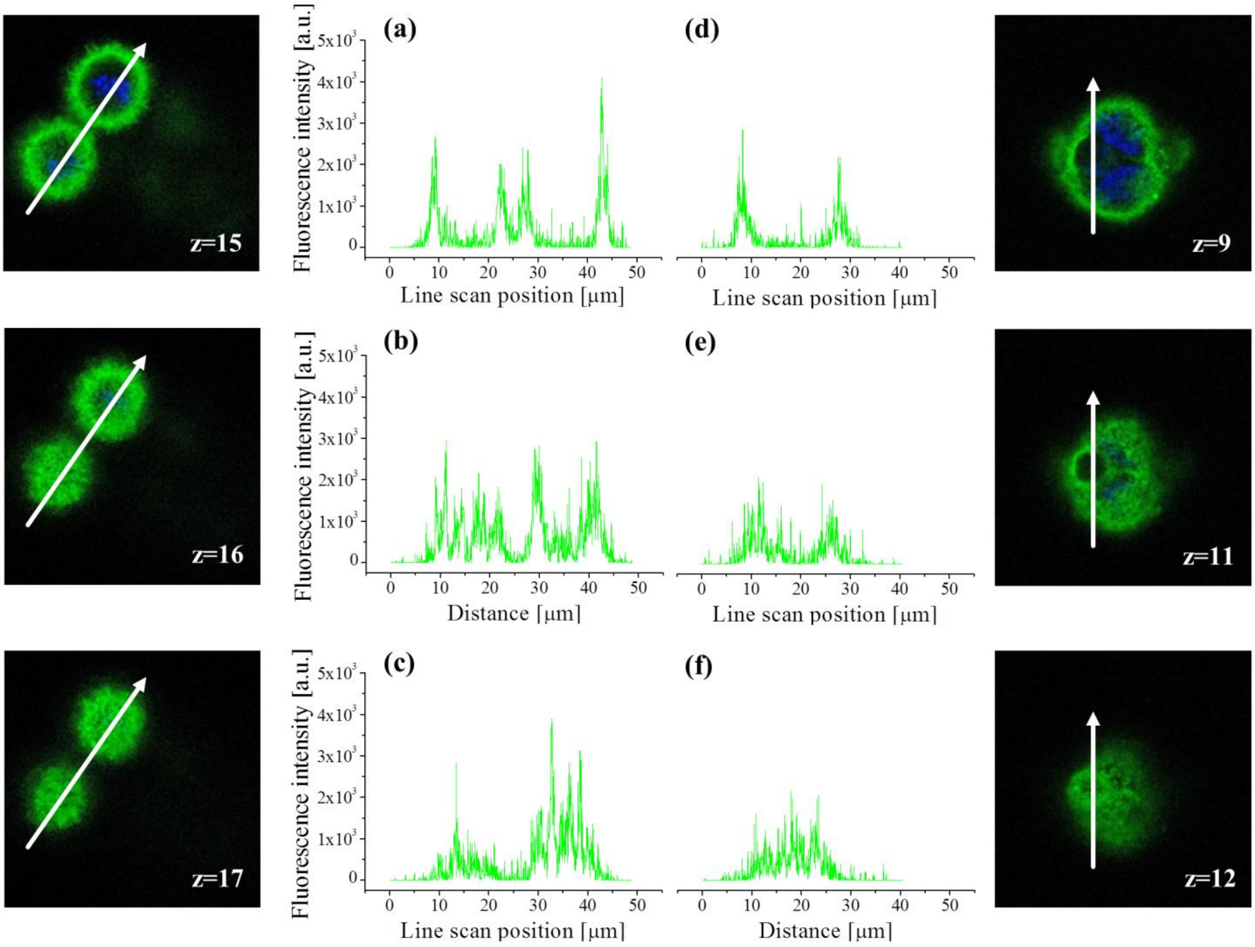

Supplementary Figure 2: Buffer solution pH effect on the yield of membrane protein biotinylation.

CFM analysis was carried out on cells treated with Alexa-SA/NHS-B in isotonic buffer solution at different pH. From each reported image, we calculated representative fluorescence intensity profiles from in-plane line scans, which are depicted with white arrows. In (a)-(c) are shown the intensity plots obtained from the fluorescence images on the left, which were collected on cells treated at pH = 8.0 at different *z*-position of the focal plane (*z* = 0 is the position of the glass bottom dish). The length of the arrows is 50 μm. Similarly, (d)-(f) report results from a cluster of cells treated at pH = 7.6 (fluorescence images on the right, length of the arrows: 40 μm).

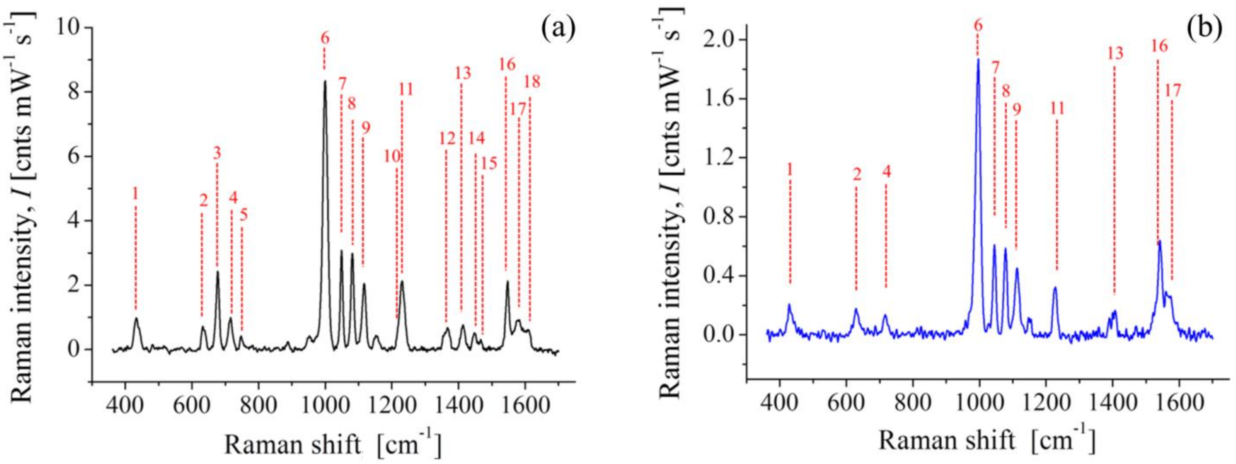

Supplementary Figure 3: Comparison between spectral features of HPDP-B and 2-Py.

Typical SERS spectra of HPDP-B (a) and 2-Py thiolate (b) collected from AuNP colloidal solutions after conjugation. The assignments of the labelled bands are reported in *Supplementary Table 1*.

Band number	SERS band position [cm^{-1}]	Band assignment	Reference
1	436	β(CCC), δ(C–S) - 2Py	[1]
2	636	6a, γ(CCC) - 2Py	[1]
3	680	ν(C–S) - BH	[4]
4	717	ν(C–S) - 2Py	[1]
5	747	ν(C–S) - BH	[4]
6	1001	1a, Ring breathing - 2Py	[1],[2]
7	1051	18a, β(C–H) - 2Py	[1], [2]
8	1081	18b, δ(C–H) - 2Py	[1]
9	1116	12a, Ring breathing/ν(C–S) - 2Py	[1],[2]
10	1220	Ureido ring+δ(CH_2) – BH	[3]
11	1229	γ(NH)/δ(NH) - 2Py	[1]
12	1367	δ_ω(CH_2) – BH	[5]
13	1414	19b, ν(C=C)/ν(C=N) - 2Py	[1]
14	1448	δ_s(CH_2) – BH	[3]
15	1466	δ_s(CH_2) – BH	[3]
16	1546	8b, ν(C=C) - 2Py	[1],[2]
17	1579	8a, ν(C=C) - 2Py	[1]
18	1609	δ_s(CH_2) - BH	[3]

γ=out-of-plane deformation; β= deformation; ν=stretching; δ_ω=wagging; δ_s=scissoring; 2Py=2-pyridine thiolate; BH=biotin-hexyl spacer arm thiolate

Supplementary Table 1: Band assignments of the spectra in Supplementary Figure 3.

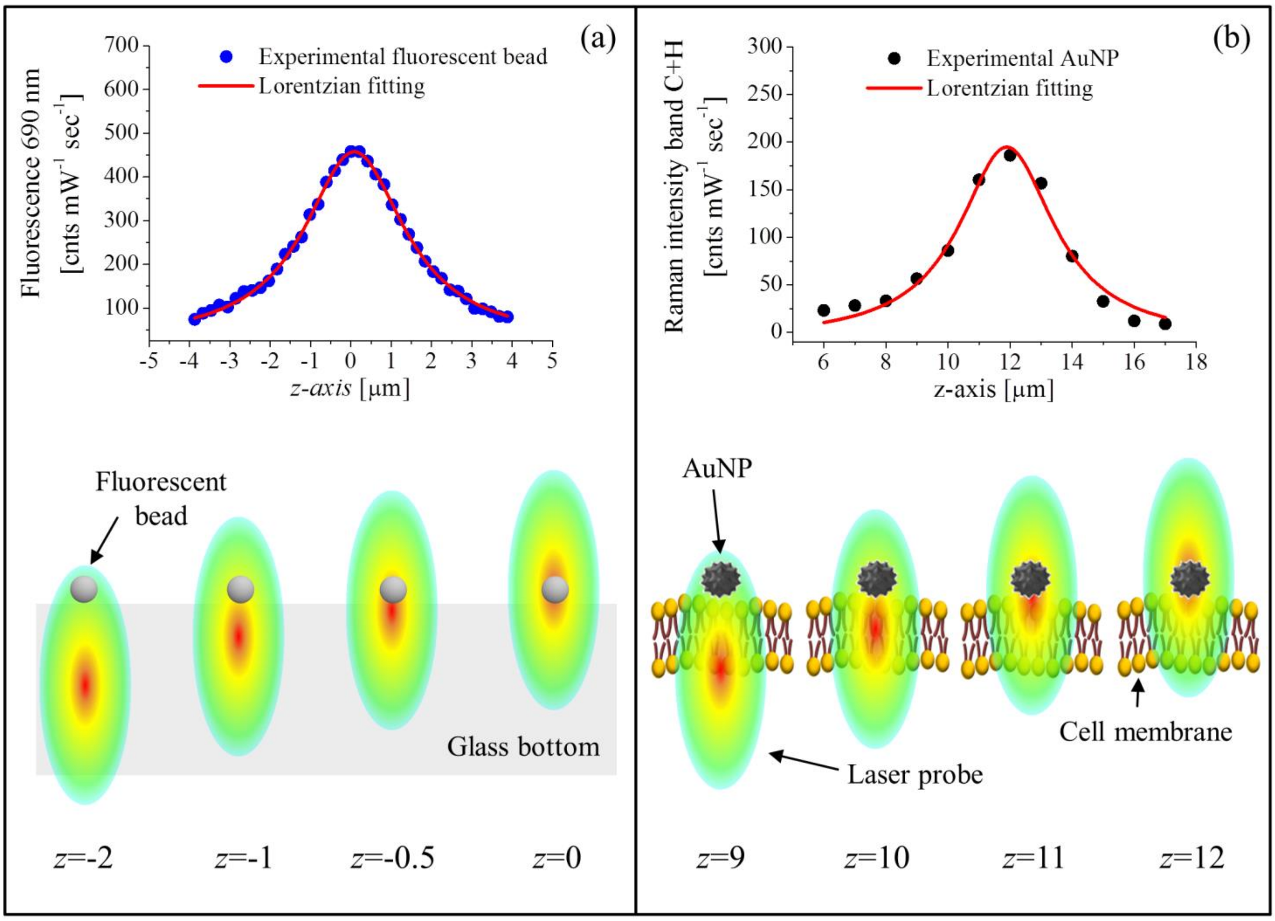

Supplementary Figure 4: Axial resolution of the laser probe estimated using fluorescence spectroscopy and SERS.

Fluorescence (a) and SERS (b) intensities profiles were obtained from *z*-axis line scans through one 50 nm fluorescent bead attached to the glass substrate and through the AuNP of *Fig. 8* in the manuscript, respectively. The experimental trends were fitted using Lorentzian function describing the intensity axial profile of the laser probe. The FWHM estimated from the data in (a) was 3.1 μm, while in (b) was 3.8 μm.

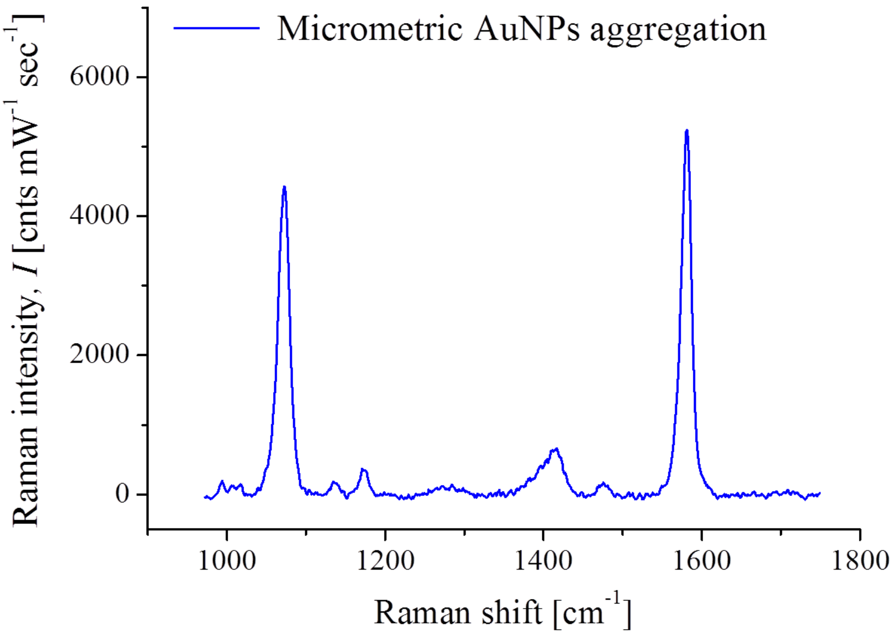

Supplementary Figure 5: Typical SERS spectrum collected from microscopic aggregations.

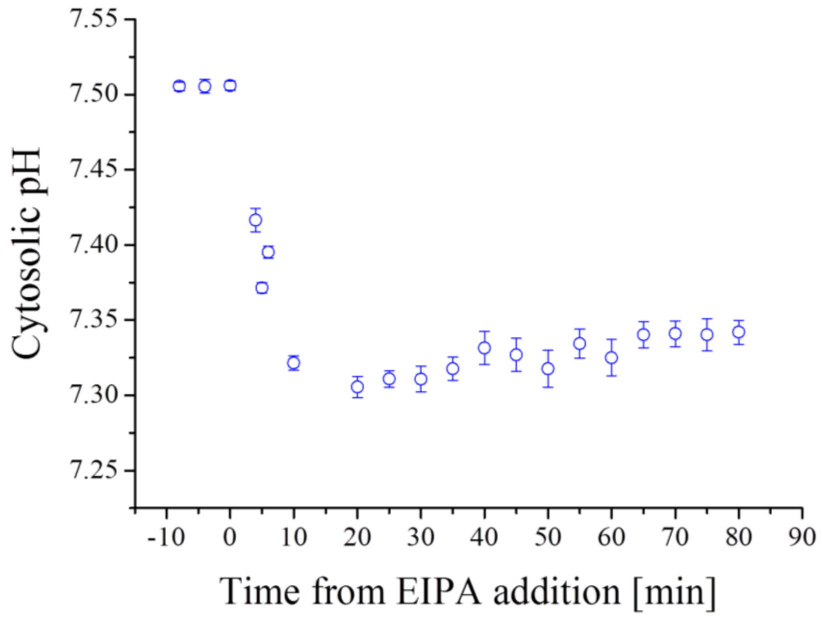

Supplementary Figure 6: Effect of EIPA on the average cytosolic pH measured in MKN28 cells.

Cytosolic pH was measured by fluorescence spectroscopy after internalization of a pH-sensitive dye. Measurements were collected at different time before and after addition of EIPA (error bars show the standard deviation from the mean of $n = 4$ measurements). Source data are provided as a Source Data file.

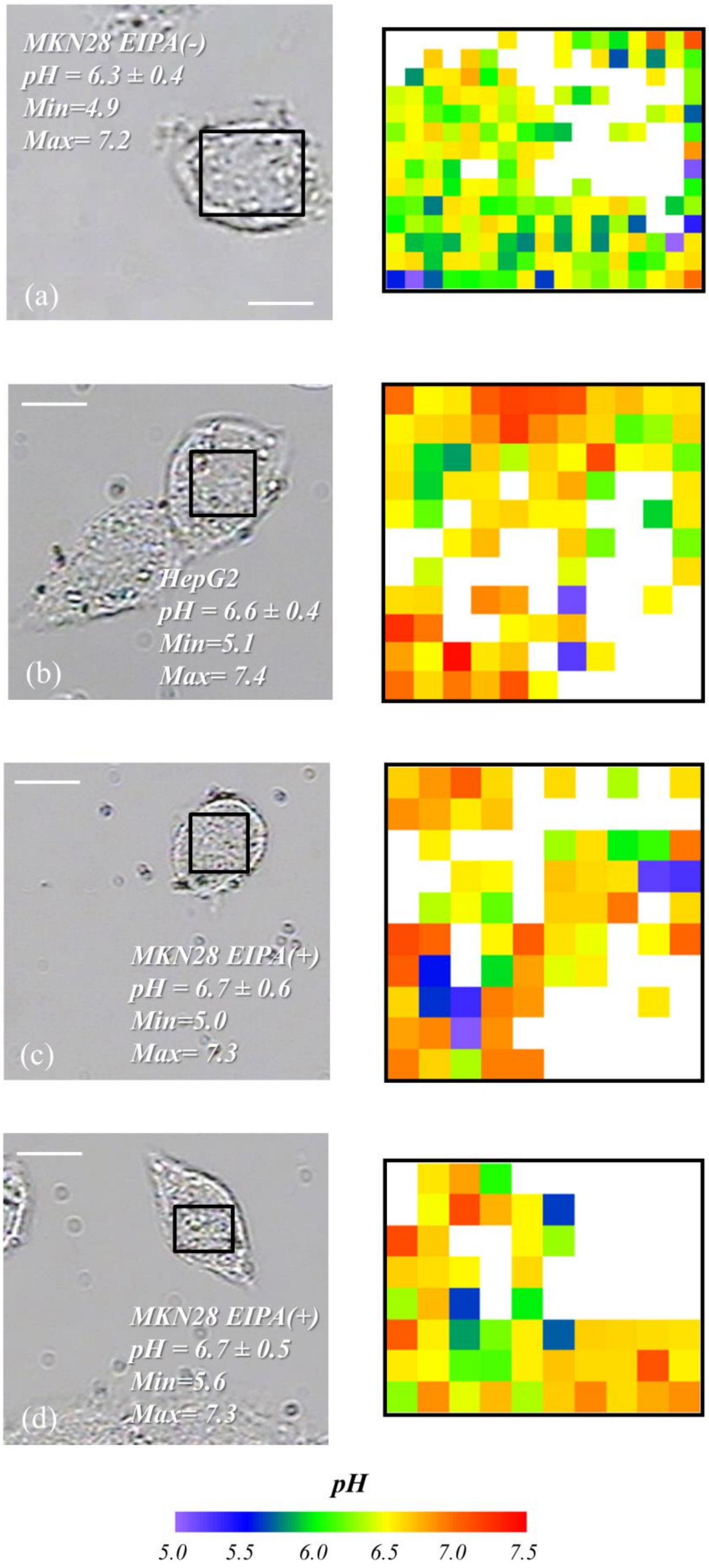

Supplementary Figure 7: Further examples of hyperspectral maps of cell surface pH.

The results were obtained from MKN28 (a), HepG2 (b) and MKN28 treated with EIPA (c)-(d). Scale bars: 10 μm.

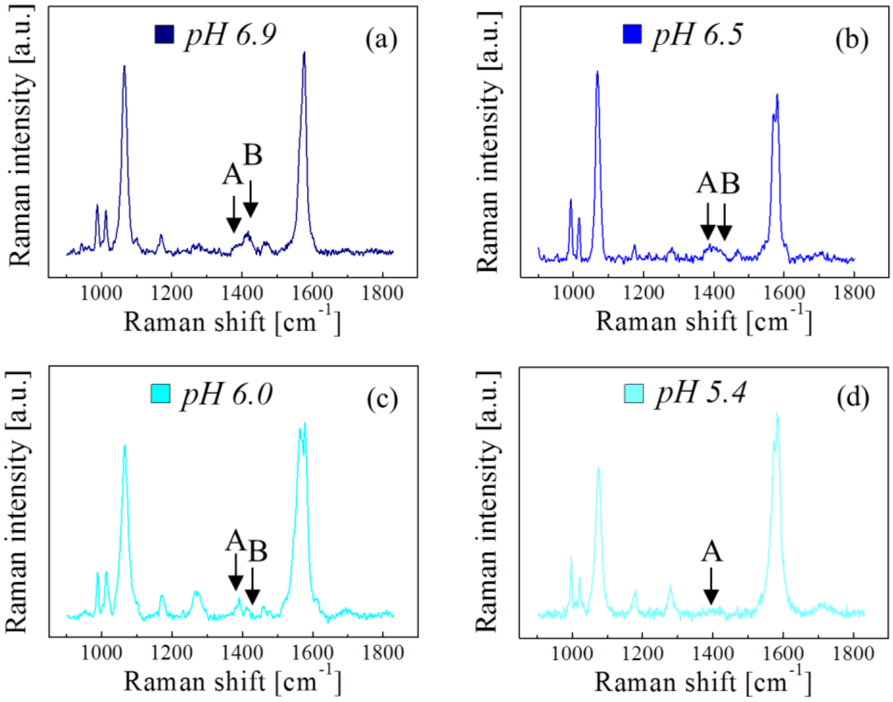

Supplementary Figure 8: Typical SERS spectra collected from cell surface.

Example spectra obtained from the analyses of MKN28 cells treated with 4-MBA conjugated AuNP and representative of locations at different local surface pH. At pH = 6.9 (a) and 6.5 (b), the contribution of band B to the total intensity of COO^- symmetric stretching is still strong. Conversely, at pH 6.0 (c) and 5.4 (d), the weak intensity is mainly represented by band A. (a) and (d) are also reported in Fig. 9 (g) - (h) of the manuscript.

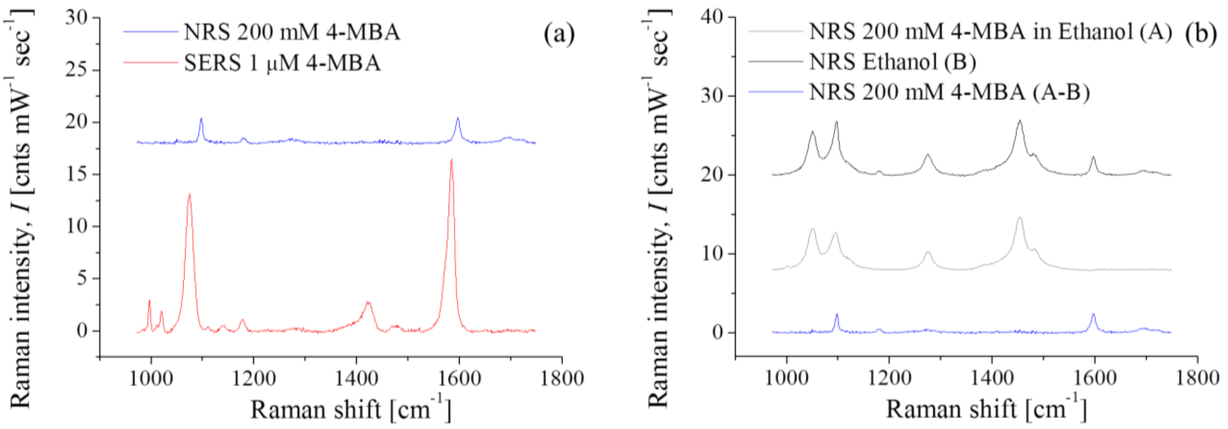

Supplementary Figure 9: Raman spectra for the calculation of the enhancement factor.

(a) Normal Raman spectrum (NRS) of 200 mM 4-MBA in ethanol solution compared to SERS spectrum from 1 µM 4-MBA colloidal solution of AuNP. (b) NRS spectrum of 4-MBA in ethanol solution shown in (a) was obtained after subtraction of the ethanol spectrum.

References:

[1]Baldwin, J. A., Vlčková, B., Andrews, M. P. & Butler, I. S. Surface-Enhanced Raman Scattering of Mercaptopyridines and Pyrazinamide Incorporated in Silver Colloid− Adsorbate Films. Langmuir 13, 3744-3751 (1997).

[2]Nalbant Esenturk, E. & Hight Walker, A. Surface - enhanced Raman scattering spectroscopy via gold nanostars. Journal of Raman Spectroscopy: An International Journal for Original Work in all Aspects of Raman Spectroscopy, Including Higher Order Processes, and also Brillouin and Rayleigh Scattering 40, 86-91 (2009).

[3]Fraire, J. C., Pérez, L. A. & Coronado, E. A. Cluster size effects in the surface-enhanced Raman scattering response of Ag and Au nanoparticle aggregates: experimental and theoretical insight. The Journal of Physical Chemistry C 117, 23090-23107 (2013).

[4]Podstawka, E., Ozaki, Y. & Proniewicz, L. M. Part III: Surface-enhanced Raman scattering of amino acids and their homodipeptide monolayers deposited onto colloidal gold surface. Applied spectroscopy 59, 1516-1526 (2005).

[5]Smith, E. A. et al. Formation, spectroscopic characterization, and application of sulfhydryl-terminated alkanethiol monolayers for the chemical attachment of DNA onto gold surfaces. Langmuir 17, 2502-2507 (2001).
