# Supplementary material for: Bioconjugation strategy for cell surface labelling with gold nanostructures designed for highly localized pH measurement
Source: Nat Commun. 2018 Dec 11;9:5278. doi: 10.1038/s41467-018-07726-5 (PMC6290020; doi:10.1038/s41467-018-07726-5)
Supplement: Supplementary file 5 — Description of Additional Supplementary Files [file 41467_2018_7726_MOESM5_ESM.docx]

Description of Additional Supplementary Files

**Supplementary Movie 1:** Movie of the z-stack fluorescence images collected through the cells of Fig. 2 after anchoring AuNP conjugated with Alexa fluorescence dye (green). Nuclei are stained with Hoechst 33342 dye (blue).

**Supplementary Movie 2:** Movie of the z-stack fluorescence images collected through the cells of Supplementary Figure 1 after labelling the surface proteins with NHS-B conjugated to Alexa fluorescence dye (green). Nuclei are stained with Hoechst 33342 dye (blue).
